# Correlation of coronary artery calcium score and carotid artery intima-media thickness with severity of coronary artery disease

**DOI:** 10.34172/jcvtr.2020.14

**Published:** 2020-05-04

**Authors:** Sudhakar Reddy Pathakota, Rajasekhar Durgaprasad, Vanajakshamma Velam, Lakshmi AY, Latheef Kasala

**Affiliations:** ^1^Department of Cardiology, Sri Venkateswara Institute of Medical Sciences, Tirupati, Andhra Pradesh, India; ^2^Department of Radiology, Sri Venkateswara Institute of Medical Sciences, Tirupati, Andhra Pradesh, India

**Keywords:** Coronary Artery Calcium Score, Carotid Artery, Intima-Media Thickness, Coronary Artery Disease

## Abstract

***Introduction:*** Coronary artery calcium score (CACS) and carotid artery intima-media thickness (CIMT) are the markers of atherosclerosis. An association between CACS and CIMT with presence of atherosclerotic coronary artery disease (CAD) is well established. However relationship between severity of CAD with CACS and CIMT is less clear. This study aimed to investigate the correlation between severity of CAD assessed by SYNTAX and Gensini scores with CACS and CIMT.

***Methods:*** This prospective study was conducted on 351 patients with CAD between June 2015 to December 2016. CACS was obtained using AGATSTON algorithm with 128 slice multidetector computer tomography (MDCT) before conventional coronary angiography (CCA). CIMT was measured by using Philips IE33 Echo machine. The severity of CAD was assessed by SYNTAX score (SS) and Gensini score on CCA. Correlation between severity of CAD with CACS and CIMT was analysed.

***Results:*** Mean CACS was 241.5±463.7, and this was positively correlated with over all SS (r=0.417, *P* <0.0001) and Gensini score (r=0.405, *P*<0.0001). Mean CIMT was 0.80±0.18 mm and this was also significantly correlated with SS (r=0.450, *P*<0.0001) and Gensini score (r=0.459,<0.0001). Multivariate analysis showed that CACS was independently associated with diabetes mellitus (β:0.11, *P*=0.021), SS (β:0.251, *P* =0.010) and mean CIMT (β:0.128, *P* =0.028). Receiver-operating characteristic (ROC) curve analysis revealed a cut off CACS of >493 for SS≥33 (high-SS tertile).

***Conclusion:*** Our study confirmed a significant correlation between CACS and CIMT with the severity of CAD assessed by SS and Gensini scores. CACS and CIMT may be considered as important noninvasive diagnostic modalities in the assessment of the severity of CAD.

## Introduction

Severity and complexity of coronary artery disease (CAD) have recently attracted increasing interest for treatment and prognostication. Studies have demonstrated that the severity and complexity of CAD assessed by Gensini score and Syntax score (SS), respectively, are associated with increased cardiovascular events a factor of morbidity and mortality.^1,2^ Therefore, efforts have been made for the prediction of CAD severity and complexity using non-invasive methods in order to identify the patients at high risk for cardiovascular events.^3,4^

Coronary artery calcium score (CACS) and carotid artery intima-media thickness (CIMT) are established surrogate markers of atherosclerosis. They predict future cardiovascular events, however it remains unclear whether they can predict the severity of CAD.

The present study aims to assess the relationship between CACS and CIMT with severity of CAD through SS and Gensini scores.

## Material and Methods

Consecutive patients (n=351) with symptomatic CAD were enrolled. Baseline observations pertaining to the clinical and demographic variables were collected from all the patients. Two-dimensional transthoracic echocardiographic (2D TTE) examination was performed with S5-1 transducer using Philips iE33 echocardiographic machine (Philips, Eindhoven, Netherlands). Measurements were captured as per the American Society of Echocardiography guidelines.^5^ CIMT was measured using L11-3 MHz linear transducer attached to echocardiographic machine.

They underwent CACS with 128 slice multi detector computer tomography (MDCT) using AGATSTON algorithm (SOMATOM DEFINITION AS+, SIEMENS Healthcare Germany) before conventional coronary angiography (CCA). Patients were excluded if they had contrast allergy, impaired renal function, arrhythmia, hemodynamic instability, congenital heart diseases, genetic disorders, post coronary artery bypass grafting (CABG) and post percutaneous coronary intervention (PCI).

### CACS measurement

CACS was performed by using 128-slice MDCT scanner (SOMATOM DEFINITION AS+, SIEMENS Healthcare, Germany) in a longitudinal scan field from tracheal carina down to the diaphragm. MDCT scan for CACS was performed by retrospective gating with collimation (4×3.0 mm) with 3-mm reconstructed slice thickness. Tube current and tube voltage were 35 mA and 120 kV, respectively and gantry rotation time was 0.4 seconds.

CACS was calculated by using Agatston method with dedicated software (SYNGO calcium score). Calcium scoring based on the Agatston method was defined as the presence of a lesion with an area >1 mm^2^, and peak intensity ≥130 Hounsfield units, which was identified and marked with color by the software automatically. All lesions were added to calculate CACS by the Agatston method.

### Coronary angiography

Coronary angiography was performed percutaneously through either femoral or radial artery approach with standard Judkins or Tiger catheters respectively by modified Seldinger technique using Artis Zee cardiac angiography system (Siemens, Munich, Germany). Angiographic results were interpreted visually and always analyzed in two orthogonal views. Lesions were considered significant if the stenosis was ≥50% diameter and insignificant if the stenosis was ≤50%. Severity of CAD was assessed by computer-assisted SYNergy between PCI with TAXus and cardiac surgery (SYNTAX) scoring algorithm^6^ and Gensini scores.

### Syntax score

As per the baseline diagnostic angiogram, each coronary lesion showing an obstructing stenosis ≥50% of the diameter in vessels ≥1.5 mm was scored separately, and the overall Syntax score was obtained using the Syntax score algorithm by adding all the individual values. This algorithm is available on the SYNTAX website (https://www.syntax.com). The patient Syntax scores was independently assessed by single observer who was blinded to CACS data. Syntax score was categorised as low, intermediate and high Syntax scores with scores as ≤22, 23 to 32 and ≥33 or more, respectively.

### Gensini score

In this study, CAD severity was also assessed by Gensini score, which is based on the percentage of luminal narrowing. The grades of coronary artery lumen narrowing is defined as follows: score-1 for ≤25% narrowing, score-2 for 26–50% narrowing, score-4 for 51–75% narrowing, score-8 for 76–90% narrowing, score-16 for 91–99% narrowing, and score-32 for total occlusion. In next step, this primary score is multiplied by a factor that takes into account the importance of the position of the lesion in the coronary arterial tree (for the left main coronary artery – 5; for the proximal left anterior descending artery or proximal left circumflex artery – 2.5; for the mid-region – 1.5; for the distal left anterior descending artery - 1 and for the distal region of the left circumflex artery or right coronary artery – 1). The final Gensini score is the sum of all the lesion scores.^7^

### Carotid artery intima-media thickness assessment

Both the common carotid arteries of study patients were scanned longitudinally with an L11-3 MHz linear transducer using Philips iE33 echocardiographic machine (QLAB-IMT; Philips). The bulb dilation served as a landmark to indicate the border between the distal common carotid artery and the carotid bulb. Images of the common carotid artery were obtained from the distal portion, 1–2 cm proximal to the carotid bulb. The intima and media lines were represented by two bright echogenic lines. The measured distance from the leading edge of the first to the second echogenic line represents the intima-media thickness. At end-diastole, only far wall intima-media thickness of the distal 1-cm portion of the common carotid artery, just before bifurcation was measured using QLAB-IMT software (Philips).^8,9^

### Statistical analysis

Data was collected on predefined case record forms and transformed into MS Excel spreadsheets. Descriptive statistics including mean with standard deviation and frequencies with percentages were calculated for continuous and categorical variables respectively. Pearson’s correlation test was used to analyse the correlations between quantifiable variables. Association between dependent and other variables was ascertained by using multiple linear regression analysis. A *P* value <0.05 was considered as statistically significant. Receiver Operator Characteristics (ROC) analysis was performed to detect the cut-off value of CACS in predicting SS ≥33 (high-risk tertile). Statistical Package for Social Science Software (SPSS) for Microsoft Windows, version 20.0, (IBM Corp., Armonk, NY, USA) was used for statistical analysis.

## Results

The demographic profile of the patients studied is shown in Table 1. Among 351 study patients, 274 (79.5%) were males. Mean age of the study population was 56.2 ± 9.9 years. Single vessel, two and three vessel diseases were present in 91 (25.92%), 103 (29.34%) and 130 (37.04%) respectively, 19 (5.46%) were having insignificant disease and 8 (2.3%) showed normal coronaries. Mean CACS, mean SS and mean Gensini scores were 241.5 ± 463.7, 14.5 ± 9.7 and 54.5 ± 35.4 respectively. Mean CIMT was 0.80 ± 0.18 mm. Sixty one (18%) patients had a CACS = 0, among them three, two and single vessel diseases were present in 6 (9.5%), 14 (22.2%) and 24 (38.1%) respectively while insignificant disease and normal coronaries were present in 16 (25.4%) and 3 (4.8%) respectively. The majority of patients with CACS = 0 had single vessel disease.

**Table 1:** Baseline characteristics of the study population

**Variable**	**Mean±SD**
Age, (y)	56.2±9.9
Male, No. (%)	279 (79.5%)
BMI, kg/m^2^	23.8±3.8
LVEF (%)	49.7±10.3
SVD, No. (%)	91 (25.92%)
DVD, No. (%)	103 (29.34%)
TVD, No. (%)	130 (37.04%)
Normal coronaries, No. (%)	08 (2.3%)
Insignificant CAD	19 (5.4%)
CACS, HU	241.5±463.7
Syntax score	14.5±9.7
Gensini score	54.5±35.4
Mean CIMT (mm)	0.80±0.18
Risk factors	
Hypertension, No. (%)	172 (49.4%)
DM, No. (%)	161 (46%)
Dyslipidemia, No. (%)	99 (28.5%)
Smoking, No. (%)	152 (43.4%)
Alcoholism, No. (%)	88 (25.1%)
Family history, No. (%)	4 (1.2%)

BMI: body mass index; HU: houston units ; LVEF: left ventricular ejection fraction; SVD: single vessel disese; DVD: double vessel disease; TVD: triple vessel disease; DM: diabetes mellitus; SD: standard deviation; CACS, Coronary artery calcium score.

When SS was stratified into low (SS: ≤22), intermediate(SS: 22-32) and high (SS: ≥33) tertiles, study population with high SYTAX tertile group had highest mean CACS (*P* <0.001) (Table 2). When study population was divided into 4 groups according to CACS (Agatston score 1-100, 101< CACS≤ 400, 401< CACS≤ 1000, CACS≥1000), mild to minimal, moderate, severe and very severe respectively, we found that with increase in severity of CACS groups, there is increase in mean number of diseased vessels which is statistically significant (*P* =0.04) and mean SS is higher in the severe and very severe CACS groups which is statistically significant (*P* ≤ 0.0001; Table 3).

**Table 2:** Relation between mean CACS and syntax tertiles

**SYNTAX tertiles**	**N**	**Mean CACS±SE**	***P*** **value**
Low SS (≤22)	226	149.9±19.9	<0.001
Intermediate SS (23-32)	66	46.4±71.8	
High SS (≥33)	19	740.6±206.6	

CACS: coronary artery calcium score; SE: standard error; SS: syntax score.

**Table 3:** Relation between CACS (Agatston score) and SS

	**CACS≤100 (n=112)**	**100<CACS≤400 (n=117)**	**400<CACS≤1000 (n=43)**	**CACS>1000 (n=16)**	***P*** **value**
No. of diseased vessels	2.02±0.85	2.09±0.826	2.12±0.79	2.6±0.62	0.04
SS, mean	10.1±8.6	15.9±7.9	25.0±7.1	24.0±8.4	<0.0001

CACS: coronary artery calcium score; SS: syntax score.

When mean CIMT was compared between SYNTAX tertiles, we found a significant higher CIMT (1.01 ± 0.10 mm) in the high SYNTAX tertile group compared with other two groups (*P*  < 0.0001; Table 4)

**Table 4:** Relation between CIMT and SS

**Syntax tertiles**	**N**	**CIMT (Mean ± SD)**	***P*** **value**
Low SS (≤22)	266	0.80±0.19	<0.0001
Intermediate SS (23-32)	66	0.83±0.16	
High SS (≥33)	19	1.01±0.10	

CIMT: carotid artery intima media thickness; SS: syntax score.

### Correlation between CACS with SS and GENSINI scores

**Figure 1:**
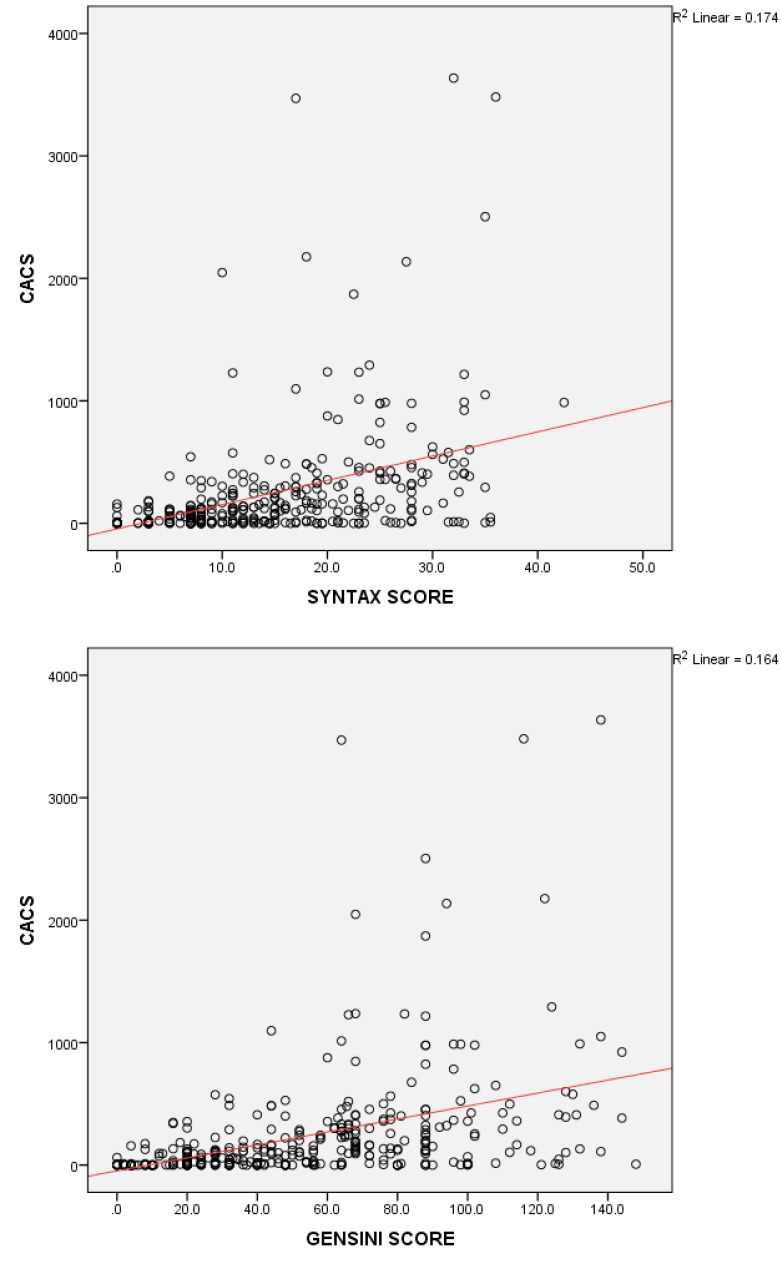
Association between CACS score with SS and Gensini score.

Pearson’s correlation analysis showed a significant correlation between CACS and SS **(**r = 0.417, *P*  < 0.0001**)** and Gensini score **(**r = 0.405, *P*  < 0.0001) respectively (Figure 1).

### Independent associates of CACS 

Multiple linear regression analysis showed that Diabetes mellitus (β =0.111 and *P* = 0.021), SS (β =0.251 and *P* = 0.010 ) and mean CIMT (β= 0.128; *P* = 0.028) were found to be independent predictors of CACS. Independent associates of CACS are shown in Table 5.

**Table 5:** Multivariate linear regression analysis showing association of CACS with other parameters

**Variables**	**Standardized beta** **(regression coefficients)**	***P*** **value**
Age	0.075	0.130
Diabetes mellitus	0.111	0.021*
SS	0.251	0.010*
Gensini score	0.155	0.107
Mean CIMT	0.128	0.028*

CIMT: carotid artery intima media thickness; SS: syntax score. Dependent variable: CACS. *indicates significant *P* value.

### Correlation Between mean CIMT with SS and GENSINI scores:

**Figure 2:**
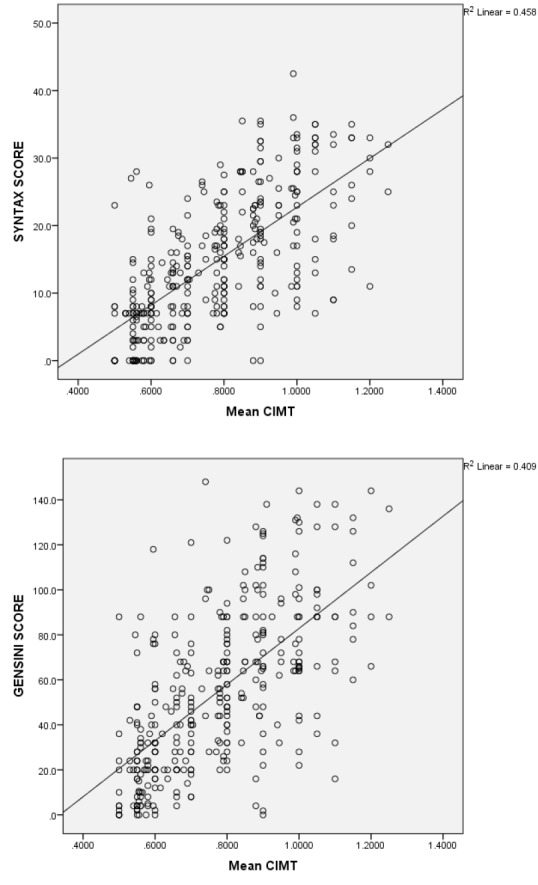
Correlation between mean CIMT and SS and Gensini score.

Pearson correlation analysis showed a significant strong and positive linear relationship between CIMT and SS **(**r = 0.450, *P* ≤ 0.0001**)**and Gensini score (r=0.459, *P* ≤ 0.0001) (Figure 2).

Among continuous variables, SS and Gensini score had strong correlation with mean CIMT (r=0.450, r=0.459 respectively, *P*  < 0.0001) whereas age and CACS exhibited weak but still significant relationship (r=0.180, *P* =0.001; r=0.250, *P*  = 0.001 respectively). On multivariate regression analysis SS and Gensini score demonstrated strongest association with CIMT among the variables.

### Assessment of cut-off point of CACS for patients with SS ≥ 33 (high SS tertile)

**Figure 3:**
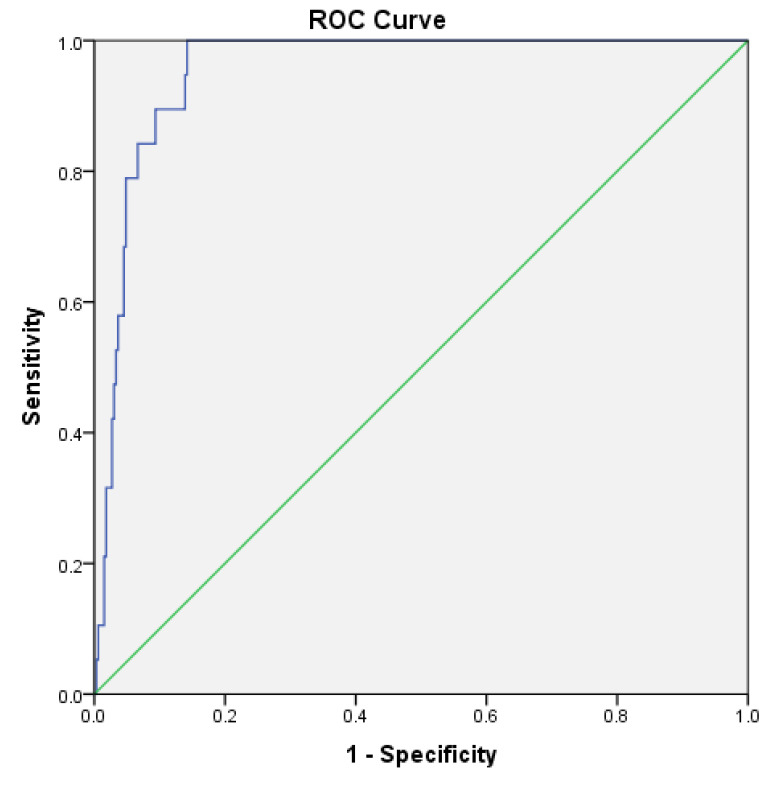
ROC curve for CACS in prediction by SYTAX score ≥33 (high risk tertile).

The cut-off value of CACS in predicting patients with SS ≥33 (high tertile) was derived using ROC curve analysis. CACS identified patients with SS ≥33 (high tertile) with specificity of 90.6 % and sensitivity of 89.5 % and with a cut-off value of >493 (AUC=0.955 and *P*  < 0.0001) (Figure 3).

## Discussion

CACS and CIMT are established surrogate markers of subclinical atherosclerosis. Large number of studies have documented their incremental value in predicting cardiovascular disease risk over conventional risk factors. Earlier studies, especially in asymptomatic patients, have considered an association between CAD and CACS. In asymptomatic patients, the prognostic value of CACS has been shown to be independent of traditional risk factors (e.g., MESA study).^10^ In symptomatic patients, the association between CACS and obstructive CAD has been demonstrated with high sensitivity and low specificity. Therefore, efforts have been made for CACS utilization as a useful filter for obstructive CAD before CCA.^11,12^ Only few studies, in symptomatic patients, have assessed the value of CACS in prediction of severity and complexity of CAD beyond prediction of obstructive CAD.^4,5^

The present study, on a per-patient basis, showed SS and Gensini scores were significantly correlating with CACS. Age, diabetes mellitus, SS, Gensini score and mean CIMT showed a relationship with CACS, among these variables, multivariate analysis revealed diabetes mellitus (β = 0.11, *P*  = 0.021), SS (β = 0.251, *P*  = 0.010) and mean CIMT (β = 0.128, *P*  = 0.028) were independently predicting CACS.

In our study, CACS was independently associated with SS and it was not independently associated with Gensini score, although it was significantly correlated. The explanations are: Gensini score depends on the percentage of luminal narrowing and coefficient of affected coronary segment. The luminal narrowing percentage had a significant impact on the scoring system and all the lesions with >25% luminal narrowing were included in the calculation. Gökdeniz et al^13^ study showed similar results on Gensini score.

There is a significant relationship between the extent of calcification and mean degree of coronary artery stenosis. In our study, out of 351 patients, 18% (n=61) had CASC=0, in them 95.08% (n=58) had coronary artery disease. Calcium is not necessarily correlated to the extent of stenosis of individual plaques, even though it is correlated to the amount of plaque. Similarly, it is not well established that the relationship between plaque calcification and the risk of plaque rupture. Recurrent subclinical episodes of plaque rupture with repeated hemorrhage and healing might constitute an important mechanism of plaque growth and worsening of luminal stenosis and might predispose plaques to calcification.^14^ However, in absence of calcium also plaques can rupture. This study results were in concordance with Henneman et al^15^ which showed that existence of vulnerable plaque does not absolutely excluded in absence of calcium.

There are few reports demonstrating a relationship between CIMT and the severity of CAD. In this study we found a significant correlation of CIMT with Syntax score (r=0.450, *P*  ≤ 0.0001) and Gensini score (r=0.459, *P*  ≤ 0.0001 ). This association of Syntax score with CIMT is in accordance with Ikeda et al study.^16^ Syntax score and Gensini scores are also independently predicting the mean CIMT.

This study provided the best cut-off value for CACS to discriminate high SYNTAX tertile from intermediate and low SYNTAX tertiles. In this regard, the cut-off was 493 for CACS, yielding acceptable sensitivity and specificity for predicting high SYNTAX tertile.

### Limitations of the study

Sample size is relatively small. Quantification of lesions was based on visual interpretation. In our study, the analysis was done based on per-patient, rather than per-vessel. Hence, CACS per-coronary artery was not included in the study. In our study for CAC scoring, only the Agatston method was used, while other methods such as mass score and calcium volume score were not included. However, most studies are based on Agatston method.

## Conclusion

Our study confirmed strong correlation of CACS and CIMT with severity of CAD assessed by SS and Gensini scores. Individuals with higher atherosclerosis burden have more complex CAD. CACS may be considered as an important non-invasive diagnostic modality in assessment of severity of CAD with relatively small radiation exposure. CACS score is independently associated with SS and diabetes mellitus. This study emphasizes that in symptomatic patients with CAD, CACS may predict cardiovascular mortality and treatment challenge before CCA.

## Competing interests

None.

## Ethical approval

This study was conducted with a prior approval from the institutional ethics committee (IEC number: 463/dt.01.06.2015) and an informed consent was obtained from all the study participants prior to the enrolment.

## References

[1] Ndrepepa G, Tada T, Fusaro M, Cassese S, King L, Hadamitzky M (2012). Association of coronary atherosclerotic burden with clinical presentation and prognosis in patients with stable and unstable coronary artery disease. Clin Res Cardiol.

[2] Farooq V, Serruys PW, Bourantas C, Vranckx P, Diletti R, Garcia Garcia HM (2012). Incidence and multivariable correlates of long-term mortality in patients treated with surgical or percutaneous revascularization in the Synergy between Percutaneous Coronary Intervention with Taxus and Cardiac Surgery (SYNTAX) trial. Eur Heart J.

[3] Gökdeniz T, Turan T, Aykan AC, Gül I, Boyaci F, Hatem E (2013). Relation of epicardial fat thickness and cardio-ankle vascular index to complexity of coronary artery disease in nondiabetic patients. Cardiology.

[4] Schmermund A, Denktas AE, Rumberger JA, Christian TF, Sheedy PF 2nd, Bailey KR (1999). Independent and incremental value of coronary artery calcium for predicting the extent of angiographic coronary artery disease: comparison with cardiac risk factors and radionuclide perfusion imaging. J Am Coll Cardiol.

[5] Gottdiener JS, Bednarz J, Devereux R, Gardin J, Klein A, Manning WJ (2004). American Society of Echocardiography recommendations for use of echocardiography in clinical trials. J A Soc Echocardiogr.

[6] Sianos G, Morel MA, Kappetein AP, Morice MC, Colombo A, Dawkins K (2005). The SYNTAX Score: an angiographic tool grading the complexity of coronary artery disease. Euro Intervention.

[7] Gensini GGMD. Chapter X. The pathological anatomy of the coronary arteries of man. In: Gensini GGMD, ed. Coronary Arteriography. Mount Kisco, New York: Futura Publishing Co.; 1975. p. 271-74.

[8] Silvestrini M, Altamura C, Cerqua R, Pasqualetti P, Viticchi G, Provinciali L (2013). Ultrasonographic markers of vascular risk in patients with asymptomatic carotid stenosis. J Cereb Blood Flow Metab.

[9] Stein JH, Korcarz CE, Hurst RT, Lonn E, Kendall CB, Mohler ER (2008). Use of carotid ultrasound to identify subclinical vascular disease and evaluate cardiovascular disease risk: a consensus statement from the American Society of Echocardiography Carotid Intima-Media Thickness Task Force Endorsed by the Society for Vascular Medicine. J Am Soc Echocardiogr.

[10] Alexopoulos N, Raggi P (2009). Calcification in atherosclerosis. Nat Rev Cardiol.

[11] Becker A, Leber A, White CW, Becker C, Reiser MF, Knez A (2007). Multislice computed tomography for determination of coronary artery disease in a symptomatic patient population. Int J Cardiovasc Imaging.

[12] Berman DS, Wong ND, Gransar H, Miranda-Peats R, Dahlbeck J, Hayes SW (2004). Relationship between stress-induced myocardial ischemia and atherosclerosis measured by coronary calcium tomography. J Am Coll Cardiol.

[13] Gökdeniz T, Kalaycıoğlu E, Aykan AÇ, Boyacı F, Turan T, Gül İ (2014). Value of coronary artery calcium score to predict severity or complexity of coronary artery disease. Arq Bras Cardiol.

[14] Burke AP, Kolodgie FD, Farb A, Weber DK, Malcom GT, Smialek J (2001). Healed plaque ruptures and sudden coronary death: evidence that subclinical rupture has a role in plaque progression. Circulation.

[15] Henneman MM, Schuijf JD, Pundziute G, van Werkhoven JM, van der Wall EE, Jukema JW (2008). Noninvasive evaluation with multislice computed tomography in suspected acute coronary syndrome: plaque morphology on multislice computed tomography versus coronary calcium score. J Am Coll Cardiol.

[16] Ikeda N, Saba L, Molinari F, Piga M, Meiburger K, Sugi K (2013). Automated carotid intimamedia thickness and its link for prediction of syntax score in Japanese coronary artery disease patients. Int Angiol.

